# Celastrol Attenuates RANKL-Induced Osteoclastogenesis *in vitro* and Reduces Titanium Particle-Induced Osteolysis and Ovariectomy-Induced Bone Loss *in vivo*

**DOI:** 10.3389/fphar.2021.682541

**Published:** 2021-06-03

**Authors:** Qiang Xu, Guiping Chen, Huaen Xu, Guoming Xia, Meisong Zhu, Haibo Zhan, Bin Zhang, Min Dai, Hongxian Fan, Xuqiang Liu

**Affiliations:** ^1^ Department of Orthopedics, the First Affiliated Hospital of Nanchang University, Artificial Joints Engineering and Technology Research Center of Jiangxi Province, Nanchang, China; ^2^ Department of Ophthalmology, the Affiliated Eye Hospital of Nanchang University, Nanchang, China; ^3^ Department of Orthopaedics, Shenzhen University General Hospital, Shenzhen University Clinical Medical Academy, Shenzhen, China

**Keywords:** osteoclast, RANKL (receptor activator for nuclear factor-κB ligand), osteolysis, osteoporosis, TAK1 binding protein 1, celastrol derivatives

## Abstract

Excessive bone resorption by osteoclasts contributes significantly to osteoclast-related diseases such as periprosthetic osteolysis and osteoporosis. Osteolysis in a titanium particle-induced calvarial model and bone loss in an ovariectomized mice model occurred similarly to those in humans; thus, these models can be used to evaluate potential therapies for aseptic prosthetic loosening and osteoporosis. Celastrol, which is extracted from the seeds of the genus Tripterygium, has been thoroughly investigated for its anti-inflammatory and anti-cancer pharmacological effects. However, the mechanisms involving bone metabolism by which celastrol inhibits osteoclastogenesis are not yet fully understood. We demonstrated that celastrol inhibited the receptor activator of nuclear factor κB ligand-induced osteoclastogenesis and the bone resorptive function of osteoclasts *in vitro* by inhibiting the activation of transforming growth factor β-activated kinase 1-mediated NF-κB and mitogen-activated protein kinase signaling pathways and downregulating osteoclastogenesis marker-related genes. Furthermore, celastrol was also shown to be beneficial in both the titanium particle-induced osteolysis calvarial and the murine ovariectomy-induced bone loss. Collectively, our results suggested that celastrol is promising for the prevention of aseptic prosthetic loosening and osteoporosis in the treatment of osteolytic diseases induced by disrupted osteoclast formation and function.

## Introduction

The skeletal system’s metabolic pattern is primarily controlled by systemic hormones or local regulating factors through the control of osteoblastic bone formation and osteoclastic bone resorption (Sims and Gooi, 2008). Disruption of the balance between bone formation and resorption leads to skeletal osteolytic disease. Osteoclasts, as the primary cells active in bone resorption, induce bone matrix deterioration and therefore play an important role in the bone metabolism mechanism (Teitelbaum, 2000). The increased number and activity of osteoclasts can result in diseases associated with excessive bone resorption, such as periprosthetic osteolysis and osteoporosis (D’Amico and Roato, 2012). Inhibiting osteoclast development is therefore a critical goal in the treatment of these diseases.

Previous research has shown that osteoclasts are drived from monocyte/macrophage lineages (Udagawa et al., 1990), and that osteoclast proliferation and maturation need two important cytokines: macrophage colony-stimulating factor (M-CSF) and receptor activator of nuclear factor-κB ligand (RANKL) (Lacey et al., 1998; Yao et al., 2002). When RANKL binds to RANK, it recruits adaptor molecules like tumor necrosis factor receptor-associated factor 6 (TRAF6), which triggers multiple downstream signaling molecules like nuclear factor-kappaB ((NF-κB) and mitogen-activated protein kinases (MAPK) (Franzoso et al., 1997; Pearson et al., 2001). Moreover, evidence suggests that transforming growth factor β-activated kinase 1 (TAK1) is referred in RANKL signaling pathways and demonstrates that the TRAF6-TAB2-TAK1 complex is required for the activation of NF-κB (Zhang et al., 2017). As these signaling mechanisms are active, transcription factors such as c-fos (Grigoriadis et al., 1994) and nucle-ar factor of activated T cells c1 (NFATc1) (Takayanagi et al., 2002) are upregulated and activated, resulting in osteoclastogenesis. Therefore, the research on the RANKL-induced osteoclast differentiation signaling axis may not only help to elucidate the pathogenesis of osteoclast-related osteolysis disease but also provide a new research target for clinical studies to further explore its therapeutic benefits. Recent studies have highlighted the increasing popularity of the potential pharmacological activities of natural compounds and their derivatives for applications in human diseases (An et al., 2016).

Celastrol, a triterpenoid compound, plays an important role in inflammatory diseases and cancer. It has been shown to have anti-inflammatory properties in animal models of multiple inflammatory diseases, including Alzheimer’s disease, arthritis, and systemic lupus erythematosus (Venkatesha and Moudgil, 2016). Futhermore, celastrol inhibits tumor cell proliferation, survival, and metastasis in multiple cancer models *in vivo* (Kannaiyan et al., 2011). Although Aymen et al. reported that IKB kinase inhibitors inhibited osteoclast formation and prevented ovariectomy-induced bone loss, only selective selection of parthenolide and BMS-345541 prevented bone loss *in vivo*, with no indication that celastrol had the same effect (Idris et al., 2010). Therefore, the effect of celastrol on bone metabolism diseases, especially for periprosthetic osteolysis and osteoporosis, remains unclear, and if there is an effect, the underlying molecular mechanism remains to be elucidated.

Consequently, to assess celastrol as a widely administered and safe clinical application, we hypothesized that celastrol has therapeutic advantages for the management of osteoclast-related osteolytic diseases. Therefore, this research investigated the roles of celastrol in osteoclastogenesis and explored the underlying mechanism by which the effects of celastrol on osteoclastic formation and function are mediated. Additionally, we investigated celastrol’s therapeutic effect in a titanium particle-induced osteolysis calvarial model as well as a murine ovariectomy-induced bone loss model.

## Materials and Methods

### Cells, Media, and Reagents

The American Type Culture Collection (ATCC; Rockville, MD, United States) provided the RAW264.7 murine macrophage cell line. Solarbio (Beijing, China) provided high glucose dulbecco’s modified eagle’s medium (DMEM). Gibco-BRL (Sydney, Australia) provided alpha modification of eagle medium (a-MEM) and fetal bovine serum (FBS). Dojindo (Kumamoto, Japan) provided cell counting kits (CCK-8). R and D (R and D Systems, Minneapolis, MN, United States) provided recombinant mouse RANKL and M-CSF. In addition, Sigma Aldrich (St Louis, MO, United States) provided the tartrate-resistant acid phosphatase (TRAP) staining kit and celastrol. Specific antibodies against extracellular signal-regulated kinase (ERK), c-Jun N-terminal kinase (JNK), p38, TAK1, nuclear factor of kappa light polypeptide gene enhancer in B-cells inhibitor alpha (IkBα), p65, phospho-ERK (Thr202/Tyr204), phospho-JNK (Thr183/Tyr185), phospho-p38 (Thr180/Tyr182), phospho-IkBα, phospho-p65, c-fos, NFATc1, and β-actin were obtained from Abcam (Cambridge, MA, United States).

### Cell Culture and Osteoclast Differentiation Assay

As previously described (Jiang et al., 2015), bone marrow monocyte/macrophage (BMM) cells were taken from the femur and tibia marrow of 4–6 week-old C57BL/6 mice and were incubated in a T75 flask in α-MEM containing 30 ng/mL M-CSF, 15% FBS, and 1% penicillin/streptomycin at 37°C, 5% CO_2_ incubator. The process usually takes 3–4 days until BMMs reaching 90% confluence. On the other hand, RAW264.7 cells were incubated in DMEM supplemented with 15% FBS and 1% penicillin/streptomycin at 37°C in a 5% CO_2_ incubator. To prepare BMMs and RAW264.7 cells for further experiments, the cells were washed three times with phosphate buffer saline (PBS) and then digested with trypsin for about 3 min. Subsequently, BMMs were seeded in a 96-well plate at a density of 1×10^4^ cells/well in α-MEM with 30 ng/mL M-CSF, 50 ng/ml RANKL, and varying concentrations of celastrol (0, 25, 50, and 100 nM). In addition, RAW264.7 cells cultured at 3×10^3^ cells/well in a 96-well plate were treated with celastrol at various concentrations (0, 25, 50, and 100 nM) in the presence of 50 ng/ml RANKL. Both were supplemented with fresh medium every 2 days until mature osteoclasts were observed. Next, the BMMs and RAW264.7 cells were fixed with 4% paraformaldehyde and stained for TRAP activity. The number of mature osteoclasts (TRAP-positive cells with ≥3 nuclei) was counted, and their spread area was measured.

### Cell Viability Assay

According to the manufacturer’s guidance, we used CCK-8 kits to determine the cytotoxicity and proliferation effect of celastrol. Briefly, BMMs were added to 96-well plates at 1×10^4^ cells/well in triplicate and cultured for 24 h in α-MEM containing 30 ng/mL M-CSF, 15% FBS, and 1% penicillin/streptomycin. The cells were then treated with varing concentrations of celastrol (0, 12.5, 25, 50, 100, 200, 400, and 800 nM) for 6, 12, 24, 48, and 96 h, respectively. Similarly, RAW264.7 cells were plated in four 96-well plates at a density of 3×10^3^ cells/well and incubated in DMEM supplemented with 15% FBS and 1% penicillin/streptomycin for 24 h. Celastrol at concentrations of 0, 12.5, 25, 50, 100, 200, 400, and 800 nM were added to the cells at the same time and the cells were subsequently cultured for 6, 12, 24, 48, and 96 h, respectively. Next, 10 µL of CCK-8 substrate was added to each well and the plate was incubated at 37°C in 5% CO_2_ for 2 h. The absorbance of each well was measured at 450 nm with an ELX800 microplate reader (Bio-Tek Instruments Inc., Winooski, VT, United States). The cell viability relative to the control group was calculated using the following formula (experimental group optimal density (OD)—blank OD)/(control group OD—blank OD).

### F-Actin Ring Immunofluorescence Assay

A sterile coverslip was first placed on the bottom of a 24-well plate. RAW264.7 cells were then seeded at a density of 4×10^5^ cells/well with glass coverslips. After complete confluency, the cells were treated with 50 ng/ml RANKL and 0, 25, 50, or 100 nM celastrol until mature osteoclasts were observed in the control wells. We then fixed the cells for 20 min at room temperature in 4% paraformaldehyde, permeabilized them for 5 min with 0.1% Triton X-100, and washed them three times with phosphate-buffered saline. F-actin was stained with tetramethylrhodamine isothiocyanate-coupled phalloidin, while the cell nuclei were stained with 4′,6‐diamidino‐2‐phenylindole. Finally, the numbers of whole F-actin rings were counted on a coverslip with ProLong Diamond Antifade Mounting medium (Invitrogen). Images were obtained using an LSM5 confocal microscope (Carl Zeiss, Oberkochen, Germany) and analyzed using Zeiss ZEN software.

### Bone Resorption Pit Assay

Bovine bone slices were placed in the bottom of a 96-well plate in triplicate. RAW264.7 cells were then plated at 3×10^3^ cells/well in the bovine bone slices in complete DMEM containing 50 ng/ml RANKL. The cells were treated with 0, 25, 50, and 100 nM celastrol for 5 days. The RAW264.7 cells detached from the bone slices were mechanically separated. Scanning electron microscope (SEM, FEI Quanta 250)was used to examine the resorption pits and ImageJ software (National Institutes of Health, Bethesda, MD, United States) was utilized to calculate the resorption pit proportion area.

### RNA Extraction and Quantitative qPCR Assay

RAW264.7 cells were seeded in 6-well plates at a density of 1×10^5^ cells/well and incubated in DMEM with 50 ng/ml RANKL. The cells were treated with various doses of celastrol (0, 50, and 100 nM) for 5 days. Total RNA was extracted using an RNAsimple Total RNA Kit (Tiangen, Beijing, China), according to the manufacturer’s instructions. cDNA was then synthesized from 1 µg of total RNA using a FastQuant RT Kit (Tiangen). Quantitative polymerase chain reaction (qPCR) was performed using the SYBR® Premix Ex Taq™ Kit (TaKaRa, Otsu, Japan) on an ABI StepOnePlus System (Applied Biosystems, Inc., Foster City, CA, United States). The following cycling conditions were used: 40 cycles of denaturation at 95°C for 5 s and extension at 60°C for 24 s. GAPDH was used as a housekeeping gene and each sample was tested in triplicate. The primer sequences of TRAP, Cathepsin K, CTR, c-fos, DC-STAMP, NFATc1, V-ATPase d2, V-ATPase a3, and GAPDH were as follows: TRAP forward 5′-CTG​GAG​TGC​ACG​ATG​CCA​GCG​ACA-3′ and reverse 5′-TCC​GTG​CTC​GGC​GAT​GGA​CCA​GA-3′; CathepsinK forward 5′-CTT​CCA​ATA​CGT​GCA​GCA​GA-3′ and reverse 5′-TCT​TCA​GGG​CTT​TCT​CGT​TC-3′; CTR forward 5′-TGC​AGA​CAA​CTC​TTG​GTT​GG-3′ and reverse 5′-TCG​GTT​TCT​TCT​CCT​CTG​GA-3′; c-fos forward 5′-CCA​GTC​AAG​AGC​ATC​AGC​AA-3′ and reverse 5′-AAG​TAG​TGC​AGC​CCG​GAG​TA-3′; DC-STAMP forward 5′-AAA​ACC​CTT​GGG​CTG​TTC​TT-3′ and reverse 5′-AAT​CAT​GGA​CGA​CTC​CTT​GG-3′; NFATc1 forward 5′-CCG​TTG​CTT​CCA​GAA​AAT​AAC​A-3′ and reverse 5′-TGT​GGG​ATG​TGA​ACT​CGG​AA-3′; V-ATPase a3 forward 5′-GCC​TCA​GGG​GAA​GGC​CAG​ATC​G-3′ and reverse 5′-GGC​CAC​CTC​TTC​ACT​CCG​GAA-3′; V-ATPase d2 forward 5′-AAG​CCT​TTG​TTT​GAC​GCT​GT-3′ and reverse 5′-TTC​GAT​GCC​TCT​GTG​AGA​TG-3′; GAPDH forward 5′-ACC​CAG​AAG​ACT​GTG​GAT​GG-3′ and reverse 5′-CAC​ATT​GGG​GGT​AGG​AAC​AC-3′.

### Western Blotting

To check which signaling pathways were affected by celastrol, RAW264.7 cells were seeded in 6-well plates at a density of 5×10^5^ cells/well. The cells were pre-treated with or without 50 nM celastrol for 4 h and then stimulated with 50 ng/ml RANKL for 0, 5, 10, 20, 30, or 60 min. Moreover, for determining the effect of celastrol on TAK1 signaling pathway, RAW264.7 cells were seeded in 6-well plates at a density of 5×10^5^ cells/well. When the cells were confluent, they were pre-treated with or without 50 nM celastrol for 4 h. Cells were then stimulated with or without 50 ng/ml RANKL for 5 min. In addition, to investigate the effect of celastrol on c-fos and NFATc1, RAW264.7 cells were treated with 50 ng/ml RANKL, with or without 50 nM celastrol for 0, 1, 3, or 5 days. The cells were then lyzed in RIPA lysis buffer (Applygen Technologies Inc., Beijing, China). The isolated cell lysates were removed by centrifugation at 12,000 g at 4°C for 10 min. According to the manufacturer’s guidance, the protein concentrations were calculated for the bicinchoninic acid protein assay kits. After the immune-purified protein was boiled in the load buffer for 5 min, it was separated by 10% sodium dodecyl sulfate-polyacrylamide gel electrophoresis and transferred to membranes. The membranes were blocked with 5% nonfat milk at room temperature for 1 h, and then incubated with specific primary antibodies and kept overnight at 4°C.The next day, the membranes were thoroughly washed with tris-buffered saline and Tween 20 (TBS-Tween) and incubated for 2 h at room temperature with the secondary antibody conjugated with IRDye. Finally, the protein bands were exposed to film for signal detection and with the help of the Odyssey V3.0 image scanning (Li-COR. Inc., Lincoln, NE, United States).

### Ti Particle-Induced Calvarial Osteolysis Model

We established a mouse calvarial osteolysis model as previously mentioned to evaluate the preventive effect of celastrol on osteolysis *in vivo* (Liu et al., 2014). The Animal Care Committee of the First Affiliated Hospital of NanChang University approved all experimental procedures. Briefly, Twenty-four healthy 8 week-old C57BL/6 mice were randomly divided into four groups with six mice in each: sham operation and injected with PBS (sham), Ti particle treated and injected with PBS (vehicle), Ti particle treated and injected with 2.5 mg/kg celastrol (low), and Ti particle treated and injected with 5 mg/kg celastrol (high). After anesthesia, a midline incision was made on the calvaria and the periosteum was separated from the calvaria. Then, at the middle suture of the calvaria, 30 mg Ti particles were embedded under the periosteum with a carrier and the incision was closed aseptically. Mice in the sham operation and vehicle groups were intraperitoneally injected with 50 µL PBS daily, while mice in the low and high celastrol groups were intraperitoneally injected with celastrol at 2.5 or 5 mg/kg/time every day for 2 weeks, respectively. The calvarial samples were collected after the mice were killed at the conclusion of the trial. Finally, Particle-induced osteolysis and histological staining were assessed by high-resolution micro-computed tomography.

### Ovariectomy-Induced Bone-Loss Model

Twenty-four C57BL/6 female mice (8 weeks of age) were kept in a particular pathogen-free laboratory for one week to test their immune response. All mice were randomly divided into four groups (*n* = 6 each group): sham operation with PBS injection (sham), ovariectomized with PBS injection (OVX), ovariectomized with 2.5 mg/kg celastrol (low), and ovariectomized with 5 mg/kg celastrol (high). Fifty microliters of the corresponding solution of each group were administered intraperitoneally once daily. The mice were killed after 4 weeks and their right femurs were removed and fixed 4% paraformaldehyde for micro-CT and histological examination.

### Micro-CT Scanning and Histological Analysis

High-resolution micro-CT (mCT80, Scanco Medical, Switzerland) was used to analyze the calvaria or right femurs. The Te scanning protocol included a 10 mm isometric resolution, 70 kV and 70 mA X-ray energy settings, and a voxel size of 10 mm in three-dimensional (3D) form. After reconstruction, we performed a trabecular bone analysis at the midline suture of the calvarium or the femoral epiphysis at the distal femur. The bone volume to tissue volume (BV/TV), trabecular number (Tb.N), trabecular separation (Tb.Sp), trabecular thickness (Tb.Th), and porosity were measured. The calvaria and distal femur were decalcified with EDTA, embedded in paraffin wax, and sliced into parts for staining to detect tartrate-resistant acid phosphatase (TRAP) activity after micro-CT scanning. In each sample, the numbers of TRAP-positive cells and osteoclast surface to bone surface area (Ocs/BS) were evaluated.

### Statistical Analysis

For data processing, IBM SPSS Statistics for Windows, version 23.0 (IBM Corp., Armonk, United States) was used. Means and standard deviations were used to express data from cell and animal studies (SD). Experiments were carried out at least three times indpendently. The comparisons between groups were analyzed using the Student’s t-test and one-way study of variance. *p* < 0.05 were deemed statistically significant.

## Results

### Non-cytotoxic Levels of Celastrol Inhibited Osteoclast Formation

**FIGURE 1:**
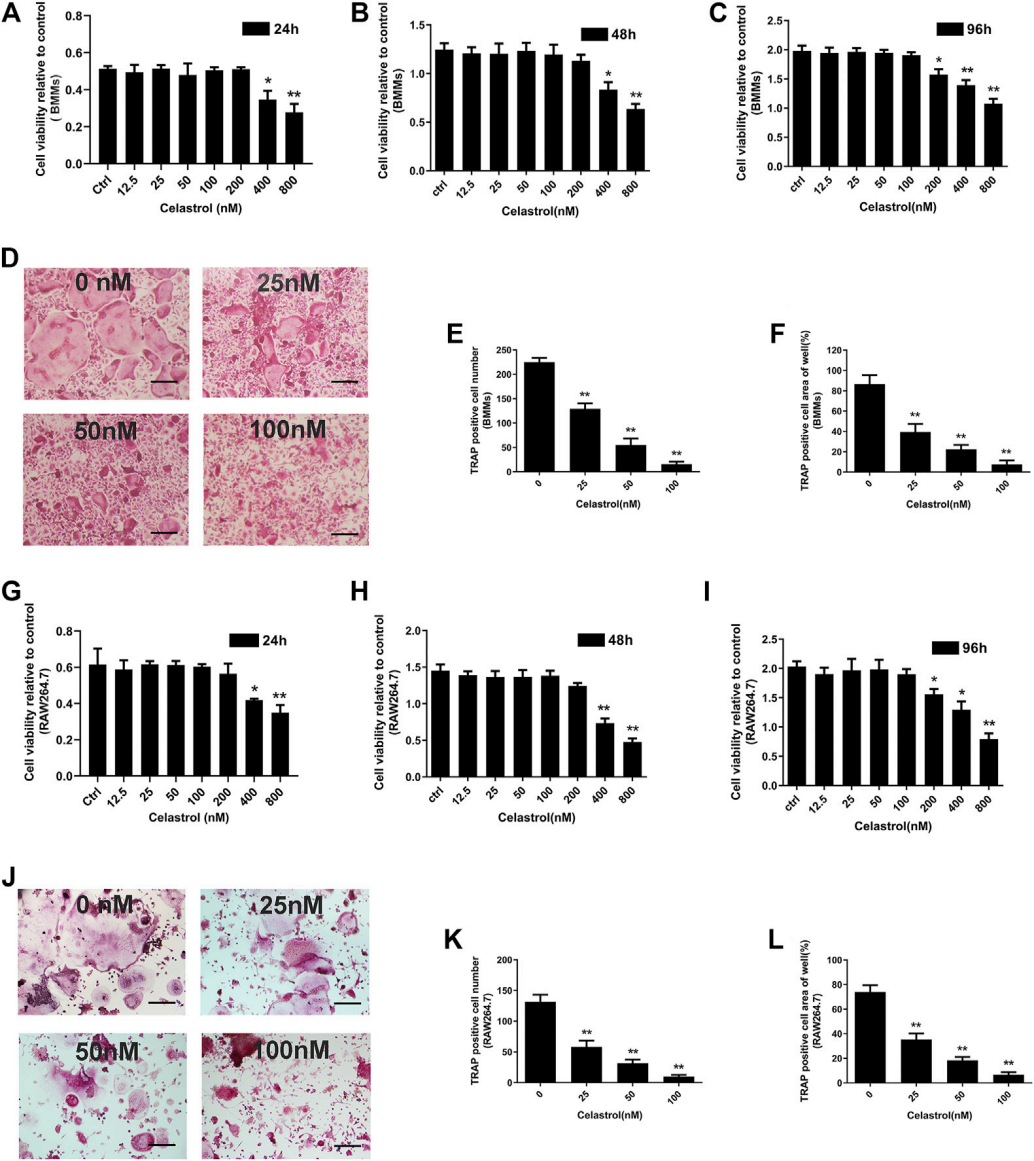
The effect of celastrol on RANKL-induced osteoclast formation had no inhibitory effect on viability in the long-term evaluation. **(A-C)** Effect of the specified concentrations celastrol on the viability of bone marrow monocytes/macrophages (BMMs) at 24, 48, and 96 h, as measured by CCK-8 assay. **(D)** BMMs were treated with 0, 25, 50, and 100 nM celastrol in the presence of 30 ng/mL M-CSF and 50 ng/ml RANKL stimulation for 5 days. The cells were then fixed with 4% paraformaldehyde and subjected to TRAP staining. Scale bar = 200 μm. **(E-F)** TRAP-positive cells (number of nuclei≥3) of BMMs cells the numbers and areas of them were quantified. **(G-I)** Effect of the specified concentrations of celastrol on the viability of RAW264.7 cells at 24, 48, and 96 h, as measured by CCK-8 assay. **(J)** RAW264.7 cells were treated with 0, 25, 50, and 100 nM celastrol in the presence of 50 ng/ml RANKL stimulation for 5 days. The cells were then fixed with 4% paraformaldehyde and subjected to TRAP staining. Scale bar = 200 μm. **(K-L)** TRAP-positive cells (number of nuclei≥3) of RAW264.7 cells the numbers and areas of them were quantified. All experiments were carried out three times, and all data are showed as the means ± SD; *p < 0.05, **p < 0.01.

To rule out that the reduction in mature osteoclast formation was due to the cytotoxicity and proliferation effects of celastrol, we investigated the effects of celastrol on BMM viability using the CCK-8 assay. Our results showed that celastrol was not cytotoxic and had no inhibitory effect on the viability of BMMs at doses <200 nM (Figures 1A–C; Supplementary Figures S1A–D). In order to investigate the effects of celastrol on osteoclast formation, we treated BMMs cultured in 30 ng/mL M-CSF and 50 ng/ml RANKL to varying concentrations of celastrol until mature osteoclasts formed. Therefore, celastrol inhibited osteoclast formation *in vitro* in a concentration-dependent manner and had no cytotoxic effect on cells. Futhermore, as indicated in Figure 1D, many TRAP-positive multinucleated osteoclasts were found in the control group in the absence of celastrol. After treatment with celastrol, the formation of TRAP-positive multinucleated osteoclasts and TRAP-positive osteoclast areas of the wells were significantly inhibited in a dose-dependent manner (Figures 1D–F). The inhibitory effect of celastrol on osteoclastogenesis was further confirmed in the RAW264.7 cell line (Figures 1G–L).

### Celastrol Inhibits F-Actin Ring Formation of Osteoclast Bone Resorption

**FIGURE 2:**
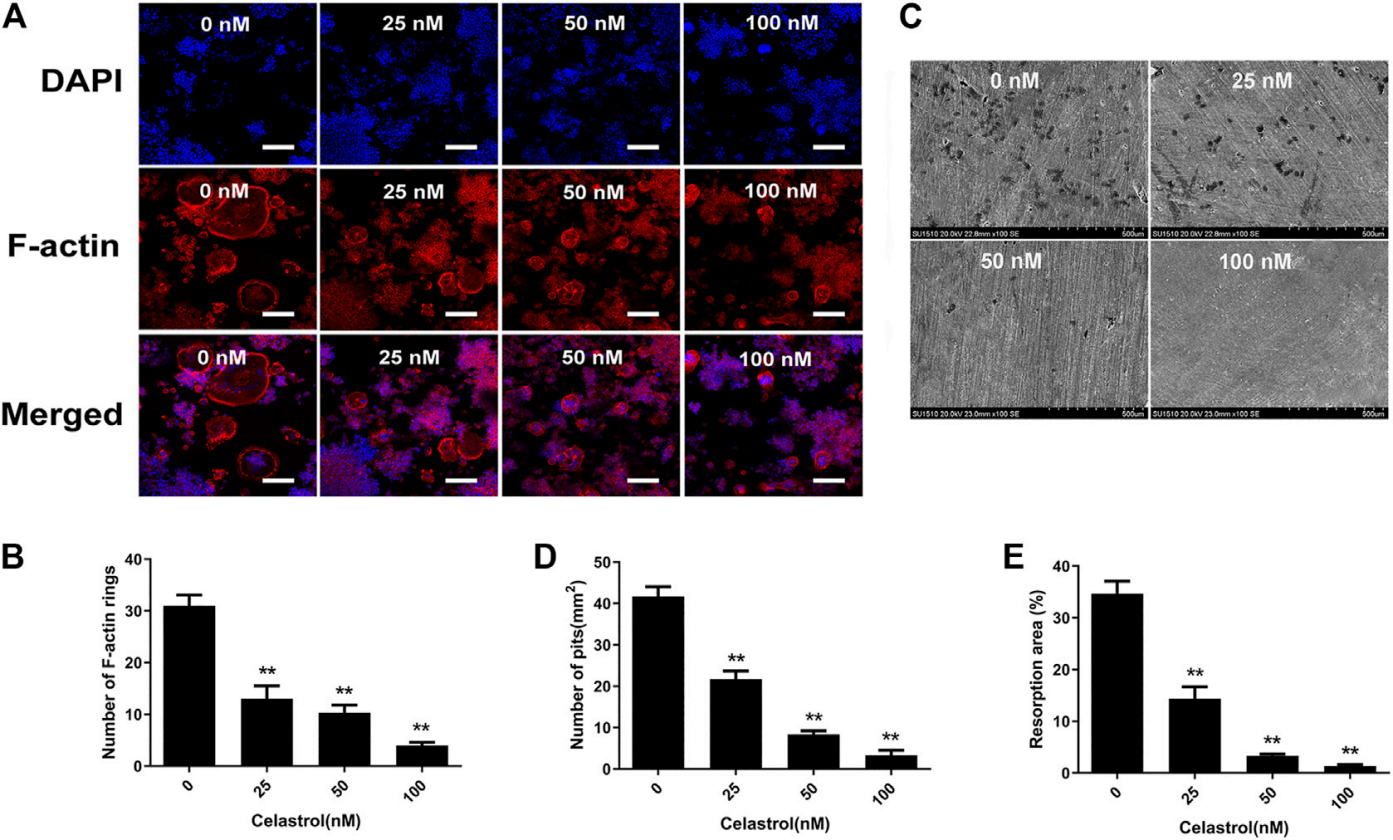
Celastrol supressed F-actin ring formation and bone resorption. Effects of celastrol on the formation of the F-actin ring. RAW264.7 cells were treated with 50 ng/ml RANKL without or with the specified concentrations of celastrol and were then fixed and immunostained for the F-actin ring. **(A)** Representative images of immunofluorescent microscopy showing the F-actin (red) and nuclei (blue) in celastrol-treated osteoclasts. Scale bar = 200 μm. **(B)** Quantification of the number of actin rings. **(C)** Effects of celastrol on osteoclastic bone resorptive function. RAW264.7 cells were stimulated with 50 ng/ml RANKL for three days. The cells were later cultured in the specified concentrations of celastrol with 50 ng/ml RANKL for another 48 h. Resorption pits were visualized by scanning electron microscopy. Scale bar = 500 μm. **(D–E)** The bone resorption (numbers and areas) for each treatment condition were quantified. All experiments were carried out three times, and all data are showed as the means ± SD; *p < 0.05, **p < 0.01.

The formation of F-actin rings and bone resorption are essential for assessing osteoclast function. Therefore, we first explored the impact of celastrol on F-actin ring formation. As seen in Figure 2A, typical F-actin ring forming was observed in untreated control group. Celastrol, on the other hand, caused dramatic changes in the number and morphology of F-actin rings in a concentration-dependent manner (Figures 2A,B). Next, to further investigate the effects of celastrol on osteoclast-mediated bone resorption, RAW264.7 cells were cultured onto bovine bone slices in medium containing RANKL until osteoclasts formed. Subsequently, these slices were treated with celastrol at different concentrations (0, 25, 50, and 100 nM) for 48 h before examination of bone pit formation by electron microscopy. As expected, the numbers and areas of bone resorption pits (darker shades) were significantly decreased compared to those in the untreated control group (Figures 2C–E). This result indicated that celastrol inhibited osteoclast formation and osteoclast function *in vitro.*

### Celastrol Inhibited Osteoclast-specific Gene Expression

**FIGURE 3:**
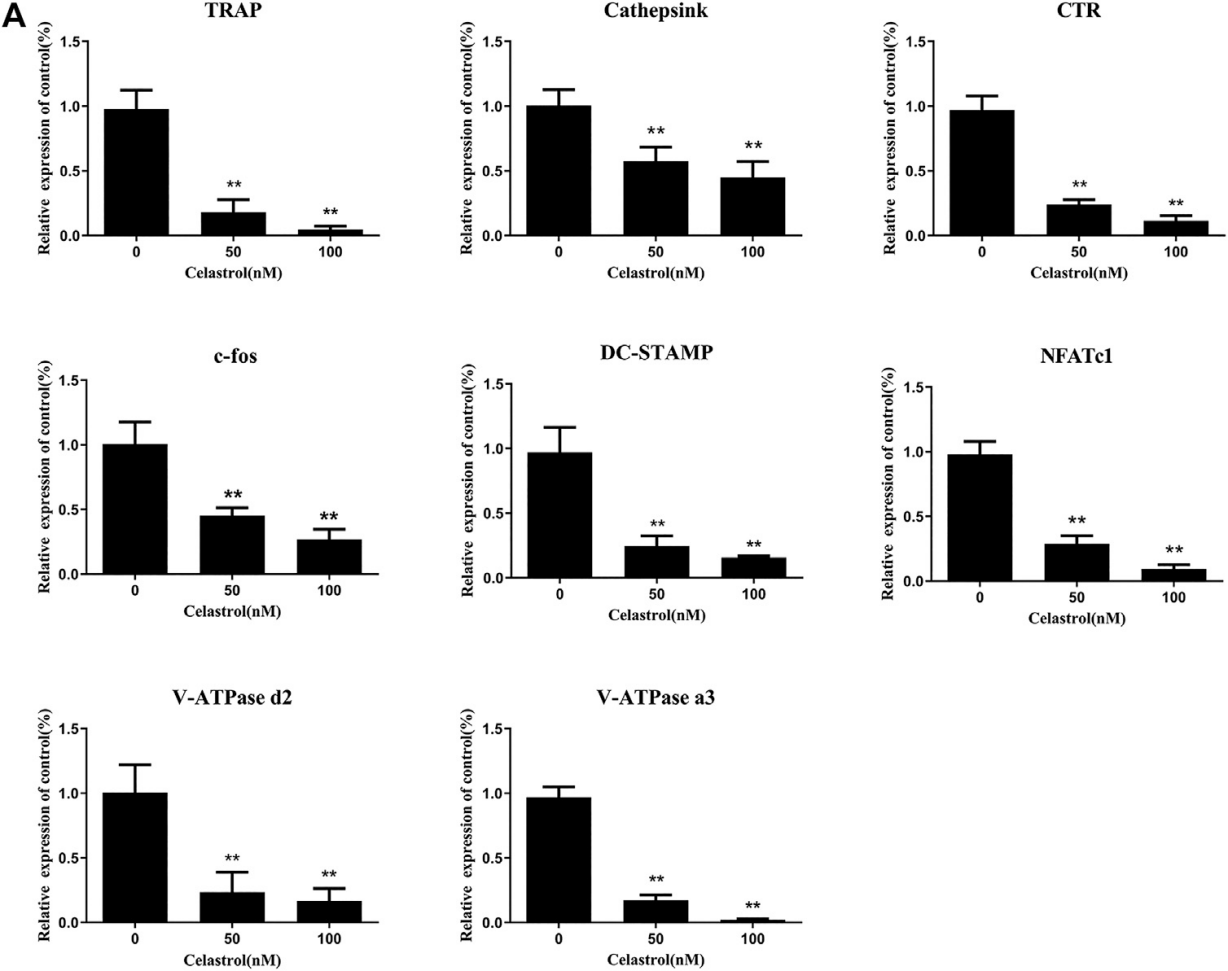
**(A)** Celastrol inhibited the RANKL-induced expression of osteoclast-specific genes. Q-PCR was applied on RNA extracted from RAW264.7 cells stimulated with 50 ng/ml RANKL and treated with 0, 25, 50, and 100 nM celastrol for 5 days. The expression levels of TRAP, Cathepsink, CTR, c-fos, DC-STAMP, NFATc1, V-ATPase d2, and V-ATPase a3 were normalized to GAPDH. All experiments were carried out three times, and all data are showed as the means ± SD; *p < 0.05, **p < 0.01.

**FIGURE 4:**
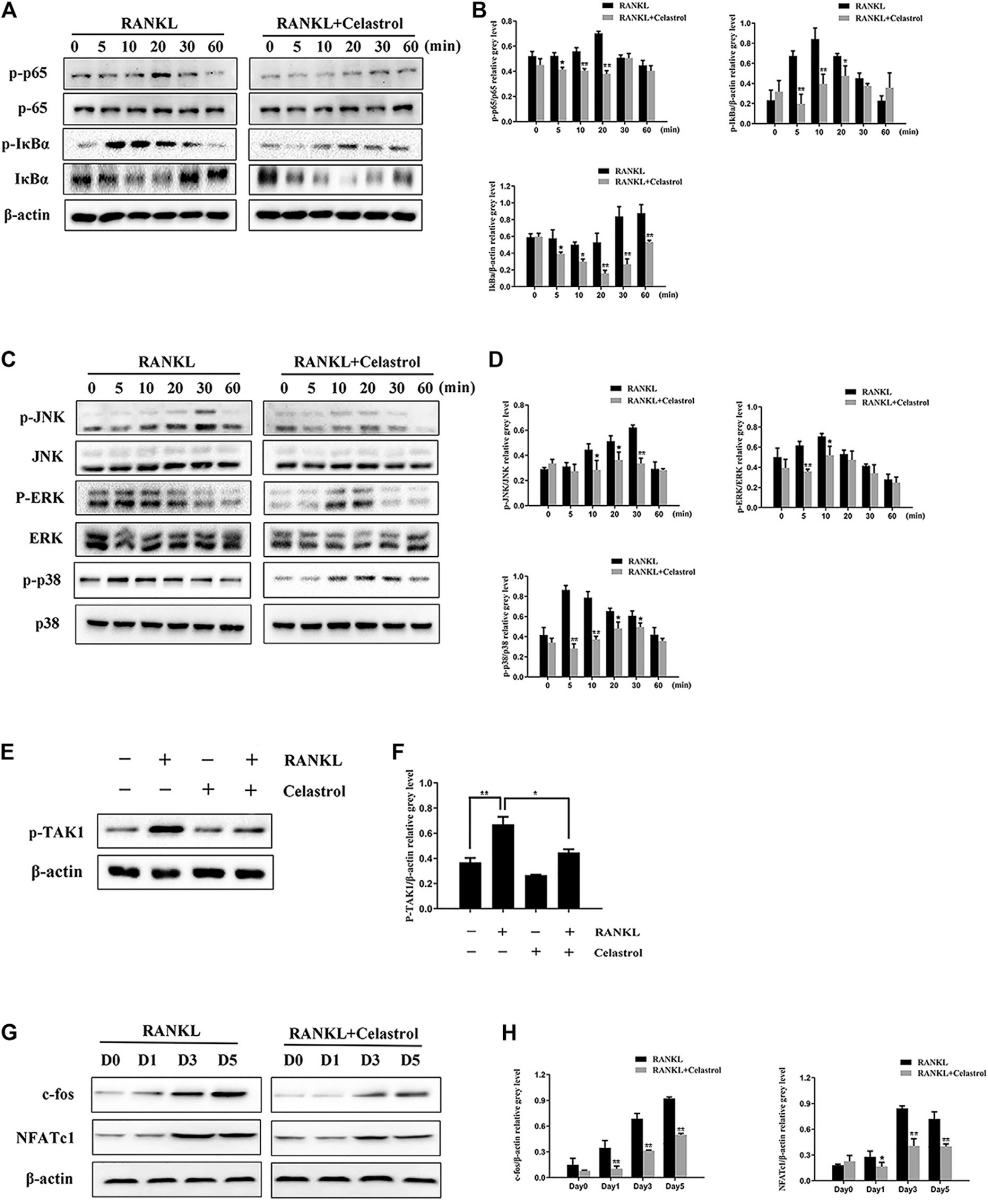
Celastrol supressed RANKL-induced activation of the TAK1-mediated NF-κB and MAPK signaling pathways. RAW264.7 cells pretreated with 50 nM celastrol or without pretreatment were stimulated with 50 ng/ml: RANKL for 0, 5, 10, 20, 30, and 60 min. The relative levels of protein phosphorylation were determined using western blot. **(A–B)** Celastrol blocked the RANKL-induced expression of the NF-κB pathway by inhibiting P65 and IκBα proteins. **(C–D)** Celastrol inhibited the phosphorylation of factors in the MAPK pathways of osteoclast formation, including ERK, JNK, and P38. **(E–F)** We also studied the upstream mechanism of the NF-κB pathway and found that celastrol inhibited TAK1 protein phosphorylation. **(G)** RAW264.7 cells were incubated in 50 ng/ml RANKL with or without 50 nM celastrol for 0, 1, 3, or 5 days, respectively. The protein expression levels of c-fos and NFATc1 were detected by western blot. **(H)** The protein expression of c-fos and NFATc1 was substantially lower in the celastrol-treated group than in the RANKL-induced group after 1, 3, or 5 days, respectively. All experiments were carried out three times, and all data are showed as the means ± SD; *p < 0.05, **p < 0.01.

To further investigate the effects of celastrol on osteoclast differentiation, levels of TRAP, Cathepsin K, CTR, c-fos, DC-STAMP, NFATc1, V-ATPase d2, and V-ATPase a3 mRNA, which are osteoclast-specific gene, were examined by quantitative PCR (qPCR). As indicated in Figure 3A, despite the fact that osteoclast-specific gene expression was dramatically upregulated in the control sample, celastrol inhibited it in a dose-dependent fashion. As a result of these findings, celastrol blocked osteoclast formation and prevented the expression of osteoclast-specific genes *in vitro*. In addition, c-fos and NFATc1 are transcription factors that promote osteoclast differentiation. As a consequence, we looked at how celastrol influenced c-fos and NFATc1 expression in RAW264.7 cells treated with RANKL and celastrol for 0, 1, 3, or 5 days. Western blot analysis revealed that RANKL triggered the expression of c-fos and NFATc1, both of which were greatly reduced by celastrol (Figure 4G). This result was followed by quantitative analysis (*p* < 0.05) (Figure 4H).

### Celastrol Suppresses TAK1-Mediated NF-κB and MAPK Signaling Pathways During Osteoclastogenesis *in vitro*

To further explore the effects of celastrol on the signaling pathways referred in osteoclastogenesis *in vitro*, we investigated the classic osteoclast signaling pathway. IκBα degradation exposes p65 to promote phosphorylation, which enables it to translocate from the cytoplasm to the nucleus to initiate the transcription of target genes. As expected after RANKL stimulation, the western blot results showed increased IκBα and p65 phosphorylation, with peaks at 10 and 20 min, respectively. However, the phosphorylation levels were significantly lower in the celastrol-treated group (Figures 4A,B). Moreover, we also studied the upstream mechanism of the NF-κB pathway and found that celastrol inhibited TAK1 protein phosphorylation (Figures 4E,F). In addition, previous studies have revealed that three major subfamilies of MAPKs (JNK, ERK, and p38) play crucial roles in osteoclast differentiation (Zhang et al., 2017). Thus, we investigated the involvement of these signaling pathways in the inhibition of osteoclast formation by celastrol. We found that celastrol significantly attenuated JNK, ERK, and p38 phosphorylation (Figures 4C,D), indicating that celastrol disrupted the MAPK pathway during osteoclast formation. Collectively, these data indicate that celastrol attenuated osteoclast formation by inhibiting TAK1-mediated NF-κB and MAPK pathways.

### Celastrol Prevented Titanium Particle-Induced Osteolysis

**FIGURE 5:**
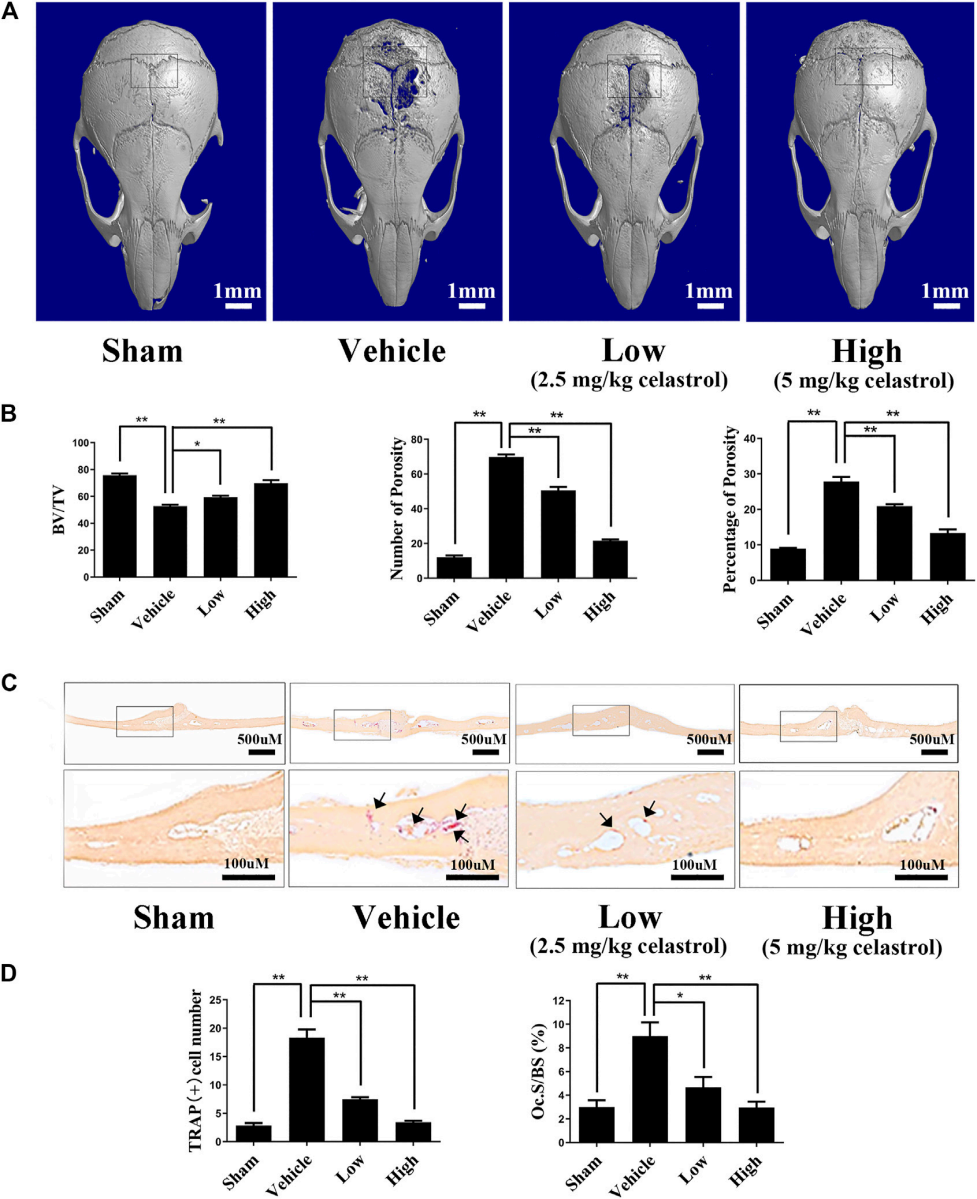
Celastrol prevented the osteolysis caused by titanium particles *in vivo.* **(A)** Representative micro-CT 3D reconstructed images were obtained for each group. **(B)** The number and percentage of porosity were calculated for each sample. **(C)** Histological assessment of Ti particle-induced mouse calvarial osteolysis with tartrate-resistant acid phosphatase (TRAP) staining. **(D)** TRAP-positive cell number and the osteoclast surface to bone surface area (Ocs/BS) were evaluated for each group. All experiments were carried out three times, and all data are showed as the means ± SD; *p < 0.05, **p < 0.01.

The *in vitro* results indicated that celastrol may reduce osteoclast-mediated osteolysis. However, the *in vivo* effects of celastrol on bone resorption of osteoclasts have not yet been determined. Therefore, we next investigated the performance of celastrol in preventing titanium-induced osteolysis in a mouse calvarial model. Compared to the sham-operated group, the vehicle-treated group had extensive erosion of the bone surface as shown by 3D micro-CT analysis (Figure 5A). Intriguingly, treatment with celastrol exhibited inhibition of titanium particle-induced osteolysis in a dose-dependent manner (Figure 5A). Moreover, quantitative analysis revealed a marked increase in BV/TV and a significant decrease in the number and percentage of bone porosity in the low and high dose groups (Figure 5B). The histological evaluation further confirmed the therapeutic effect of celastrol on titanium particle-induced osteolysis. As shown in Figure 5C, in contrast to the plentifulness of TRAP-positive osteoclasts and interrelated severe bone erosion in the vehicle group, the celastrol-treated groups showed markedly reduced numbers of TRAP-positive osteoclasts and OCs/BS (Figures 5C,D). Thus, our data demonstrated that treatment with celastrol attenuated titanium-induced osteolysis.

### Celastrol Prevented Bone Loss in Ovariectomized Mice

**FIGURE 6:**
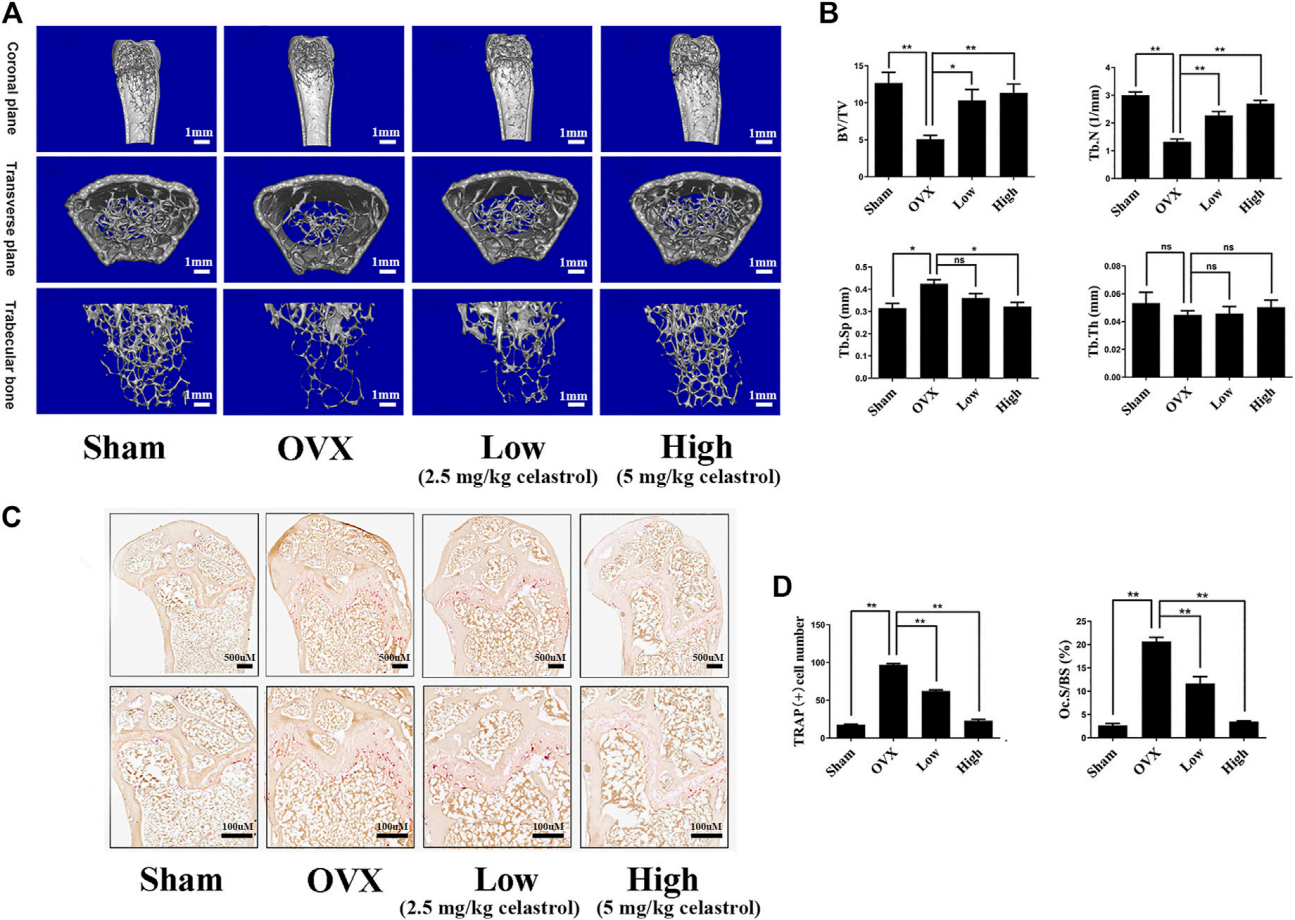
Celastrol prevented bone loss in ovariectomized mice *in vivo*. **(A)** Three-dimensional reconstruction micro-CT pictures of the femur micro-architecture. **(B)** Quantitative micro-CT examination of each sample’s bone volume to tissue volume (BV/TV), trabecular number (Tb.N), trabecular separation (Tb.Sp), and trabecular thickness (Tb.Th). **(C)** Representative images of TRAP-stained femurs. **(D)** Counting the number of TRAP-positive cells and the Ocs/BS. All experiments were carried out three times, and all data are showed as the means ± SD; *p < 0.05, **p < 0.01.

To investigate the therapeutic effects of celastrol on osteoporosis, we used the ovariectomized mouse model to assess its therapeutic potential. The mouse femurs were analyzed by micro-CT. In Figures 6A,B, compared to the sham group, the femur bone of the OVX group exhibited significant bone loss, based on drastically reduced bone volume and BV/TV, decreased Tb.N, and increased Tb. Sp. Additionally, the results showed that compared to the OVX group, the celastrol-treated mouse group had significantly reduced trabecular bone loss caused by ovariectomy in a dose-dependent manner. Furthermore, TRAP staining showed that significantly increased numbers of multinucleated osteoclasts in the OVX group but reduced numbers in the two celastrol-treated groups (Figure 6C). Conclusively, these results clarified that celastrol effectively inhibited osteoclast-mediated bone loss in osteolytic conditions*.*

## Discussion

Osteoclast-related diseases, including prosthesis osteolysis and especially osteoporosis caused by population aging, are becoming global health problems, with huge medical, economic, and social burdens. Currently, the treatments for osteoclast-related diseases are mainly anti-bone resorption drugs, such as bisphosphonates and a humanized monoclonal antibody against RANK (Miyazaki et al., 2014). Although anti-bone resorption drugs are available, their treatment results are not satisfactory. Adverse reactions such as osteonecrosis of the jaw and non-specific fractures of the femur occur frequently after long-term use of bisphosphonates (Lewiecki, 2011). In addition, while RANKL monoclonal antibodies showed an anti-bone resorption effect, clinical trials have reported the potential risks of inducing malignant tumors; thus, the drug safety requires further assessment (Goss et al., 2011; Bone et al., 2013; Bone et al., 2017). In this context, new therapeutic drugs are urgently needed for the clinical treatment of osteoclast-related osteolytic diseases. Recent studies have shown the potential use of natural medicines targeting multiple pathways in bone metabolism therapy (Hong et al., 2020; Zhu et al., 2021). Although traditional natural medicine therapies have been around for thousands of years, their active ingredients and mechanism of action remain undefined. The identification of active chemical entities and their molecular targets can reveal the clinical potential of such therapies in osteoclast-related diseases.

The results of the present study showed that celastrol protected mice against both wear particle-induced osteolysis and ovariectomy-induced bone loss through the inhibitory effect of osteoclast formation and functional activity. Concurrently, the results of our *in vitro* study showed that exposure of BMMs and RAW264.7 cells to celastrol at concentrations above 200 nM induced a cytotoxic effect according to the CCK-8 assay results. This finding suggests that a high concentration of celastrol might cause or contribute to the deaths of BMM or RAW264.7 cells. Therefore, the celastrol concentrations used in the present study (25–100 nM) aimed to exclude the celastrol induction of cytotoxic effects in the subsequent experiment. In addition, increased bone resorption capacity played a key factor contributing to osteolytic disease (Croucher et al., 2001). Our results demonstrated that celastrol significantly inhibited mature osteoclast function via the bone resorption pit assay. In line with our results, Wang’s research also found that celastrol had an inhibitory effect on osteoclast differentiation (Wang et al., 2020). Additionally, we also observed reduced F-actin formation in mature osteoclasts that was associated with impaired bone resorption activity following celastrol treatment. The effects of celastrol on these processes underscore its potential as a drug for osteoclast-related bone diseases.

Recent evidence has indicated that in the classical NF-κB pathway, the activated IκB kinase complex induces IκBα phosphorylation and degradation, which results in the release and phosphorylation of NF-κB component proteins such as p65, followed by the nuclear translocation of NF-κB and binding to a DNA target site to activate the expression of genes involved in osteoclastogenesis (Abu-Amer, 2013). Researchs have indicated that p65 gene ablation leads to the lack of osteoclast formation, thereby results in osteopetrosis, certification the indispensable role of p65 in NF-κB signaling for osteoclast differentiation (de la Rica et al., 2015). Moreover, the activation of p65 in the nucleus induces IκBα expression; moreover, the regeneration of IκBα level was positively correlated with the level of activated p65 (Sun et al., 1993). The results of Dong’s research indicated that celastrol significantly inhibited the activation of NF-κB in LPS-stimulated RAW 264.7 cells, which in turn inhibited LPS-induced inflammatory factors (Dong et al., 2017). In our study, we also found that treatment with celastrol significantly decreased the phosphorylation levels of IκBα and p65. Moreover, we also studied the mechanism by which celastrol inhibited the upstream regulation of NF-κB in the RANKL/RANK signal transduction pathway. We found that celastrol inhibited the TAK1 signaling pathway, which plays an critical role in osteoclast differentiation (Huang et al., 2006). RANKL also induced rapid phosphorylation and activation of three MAPK family members including ERK, JNK, and p38. In our study, the addition of celastrol and RANKL resulted in celastrol attenuation of the phosphorylation of these kinases in the RANKL-induced MAPK pathway. Likewise, Jung’s research also found that celastrol inhibited nitric oxide and pro-inflammatory cytokines through MAPK signaling and NF-κB in LPS-stimulated BV-2 microglial cell (Jung et al., 2007). What’s more, in diabetic rats, celastrol was found to attenuate renal injury in rats by modulating the MAPK/NF-κB pathway (Zhang et al., 2019). In dissimilarity to their report, our findings of the therapeutic effects of celastrol on osteoclast-associated bone metabolic diseases uncover these relevant pathways.

After activation, NF-κB and MAPK synergistically induce the activities of transcription factors c-Fos and NFATc1 (Takayanagi et al., 2002; Boyce et al., 2005). NFATc1 and c-fos are two important transcription factors regulating osteoclast formation in the downstream targets of NF-κB and activated protein-1 (AP-1) (Fujioka et al., 2004; Takayanagi, 2007). In a celastrol-based study of the long-term signaling pathway of osteoclast, we found significantly decreased c-fos and NFATc1 expression and protein levels following treatment with celastrol. Furthermore, the transcription factor NFATc1 has been identified as a master regulator of the expression of specific genes in the osteoclastogenesis process (Roodman, 1999; Kim et al., 2008; Feng et al., 2009). We also observed inhibited expression of the TRAP, Cathepsin K, CTR, c-fos, DC-STAMP, NFATc1, V-ATPase d2, and V-ATPase a3 genes after treatment with celastrol, thereby inhibiting bone resorption. Similarly, other reports have also found that celastrol reduced the expression of RANKL-induced osteoclast genes and transcription factors, thereby protecting against collagen-induced arthritis (CIA) in mice with bone erosion (Gan et al., 2015).

Considering the effect of celastrol *in vitro,* we further investigated its *in vivo* effects in both a titanium particle-induced osteolysis model and an ovariectomy-induced osteoporosis model. The titanium particle-induced animal osteolysis model is a well-established model for studying the characteristics and bone changes associated with the treatment of wear debris-mediated periprosthetic osteolysis. In our study, celastrol alleviated bone destruction, as reflected by the increased BV/TV and porosity in mouse calvaria. Furthermore, histological assessments were performed to verify that celastrol has an inhibitory effect by inhibiting osteoclast activity. To our knowledge, these results are the first to show the potential effectiveness of celastrol in the treatment of wear particle-induced periprosthetic osteolysis. We also used an *in vivo* osteoporosis model induced by estrogen deficiency in ovariectomized mice, which is the most common experimental method to evaluate bone microstructure changes similar to those observed in humans. Similarly, the protective effect of celastrol was demonstrated by micro-CT analysis and TRAP staining. Together, the findings of our study indicate that celastrol could provide new strategical insights for the management or treatment of osteoclast-mediated osteolytic diseases such as periprosthetic osteolysis and osteoporosis.

Currently, bisphosphonates are the classic and most widely used drugs in clinical practice for the prevention and treatment of abnormal bone metabolism (Watts and Diab, 2010). However, there are a number of adverse events or contraindications to the clinical use of bisphosphonates that limited their widespread use in the long term (Lewiecki, 2011). Compared to bisphosphonates, both celastrol and bisphosphonateare are anti-bone resorption drugs that regulate bone metabolism by inhibiting osteoclast differentiation. However, a further comparison under the same experimental conditions is needed to determine which one is more effective, but it is possible that celastrol may have lower side effects and cost advantages in the prevention and treatment of bone metabolic diseases (Xu et al., 2021). In addition, compared with the classic traditional Chinese medicine icariin, celastrol also had the inhibitory ability of RANKL-induced osteoclast differentiation. Previous studies have shown that icariin (10^4^nM) has an inhibitory effect on osteoclast activity (Chen et al., 2007; Xu et al., 2019). In the present study, our results showed that the dose of celastrol achieved significant inhibition of osteoclast differentiation at a dose of only 100 nM, suggesting that celastrol may be more effective than icariin in the treatment of bone metabolic disorders such as osteoporosis under equivalent conditions.

In this study, we observed that celastrol had an anti-osteoclastic effect, indicating its potential as an anti-aseptic prosthetic loosening treatment and possibly as an anti-osteoporosis therapy for wide application in humans. However, our study has some limitations. For instance, the impact of the systemic application of celastrol on major tissues and organs requires evaluation to determine the safety of the drug *in vivo*. In the current study, no abnormal behavior or health conditions were observed in any animals. These results support the viewpoint that the dose of celastrol was relatively safe for the treatment and prevention of osteolysis diseases.

**FIGURE 7:**
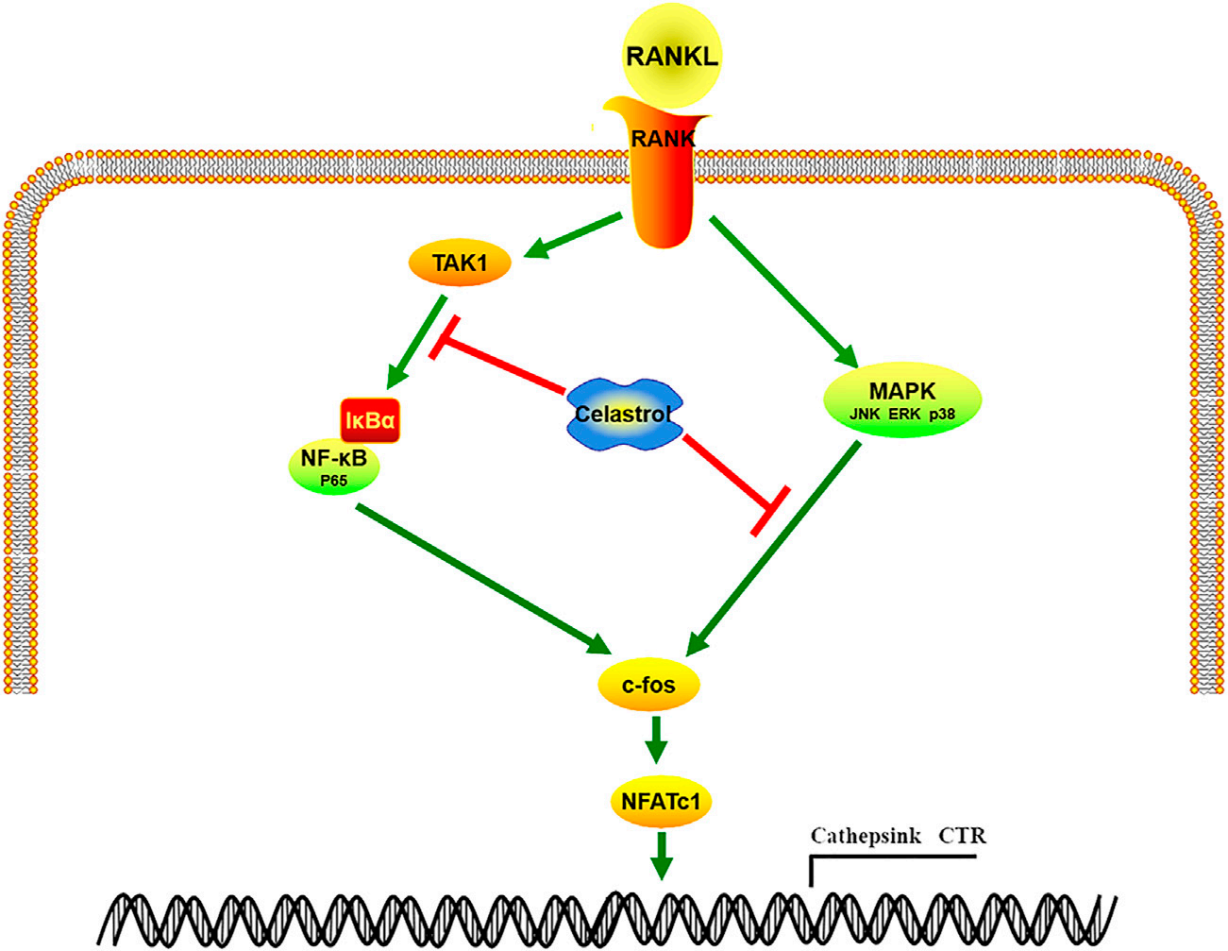
Diagram of celastrol’s inhibitory impact on osteoclast differentiation through the TAK1-mediated NF-κB and MAPK (JNK, ERK, p38) signaling pathways.

In conclusion, our findings demonstrated that celastrol inhibited osteoclast formation and function both *in vitro* and *in vivo*. Furthermore, celastrol mediated its effects through the suppression of the TAK1-mediated NF-κB and MAPK (JNK, ERK, P38) signaling pathways (Figure 7). Moreover, our *in vivo* results also showed that celastrol protected bone mass in both a wear particle-induced osteolysis model and an ovariectomy-induced bone loss murine model. Thus, our study identified celastrol as a potential new drug for the treatment of osteoclast-related bone metabolic disease.

## Data Availability

The original contributions presented in the study are included in the article/Supplementary Material, further inquiries can be directed to the corresponding authors.

## Supplementary Material

**SUPPLEMENTARY FIGURE 1** | Celastrol inhibited RANKL-induced osteoclast formation without any cytotoxicity in the short-term evaluation. **(A-D)** Effect of the specified concentrations celastrol on the viability of bone marrow monocytes/macrophages (BMMs) and RAW264.7 cells at 6,12 h, as measured by CCK-8 assay. All experiments were carried out three times, and all data are showed as the means ± SD; **p* < 0.05, ***p* < 0.01.
