# Supplementary figures and images for: Celastrol Attenuates RANKL-Induced Osteoclastogenesis in vitro and Reduces Titanium Particle-Induced Osteolysis and Ovariectomy-Induced Bone Loss in vivo
Source: Front Pharmacol. 2021 Jun 3;12:682541. doi: 10.3389/fphar.2021.682541 (PMC8210420; doi:10.3389/fphar.2021.682541)

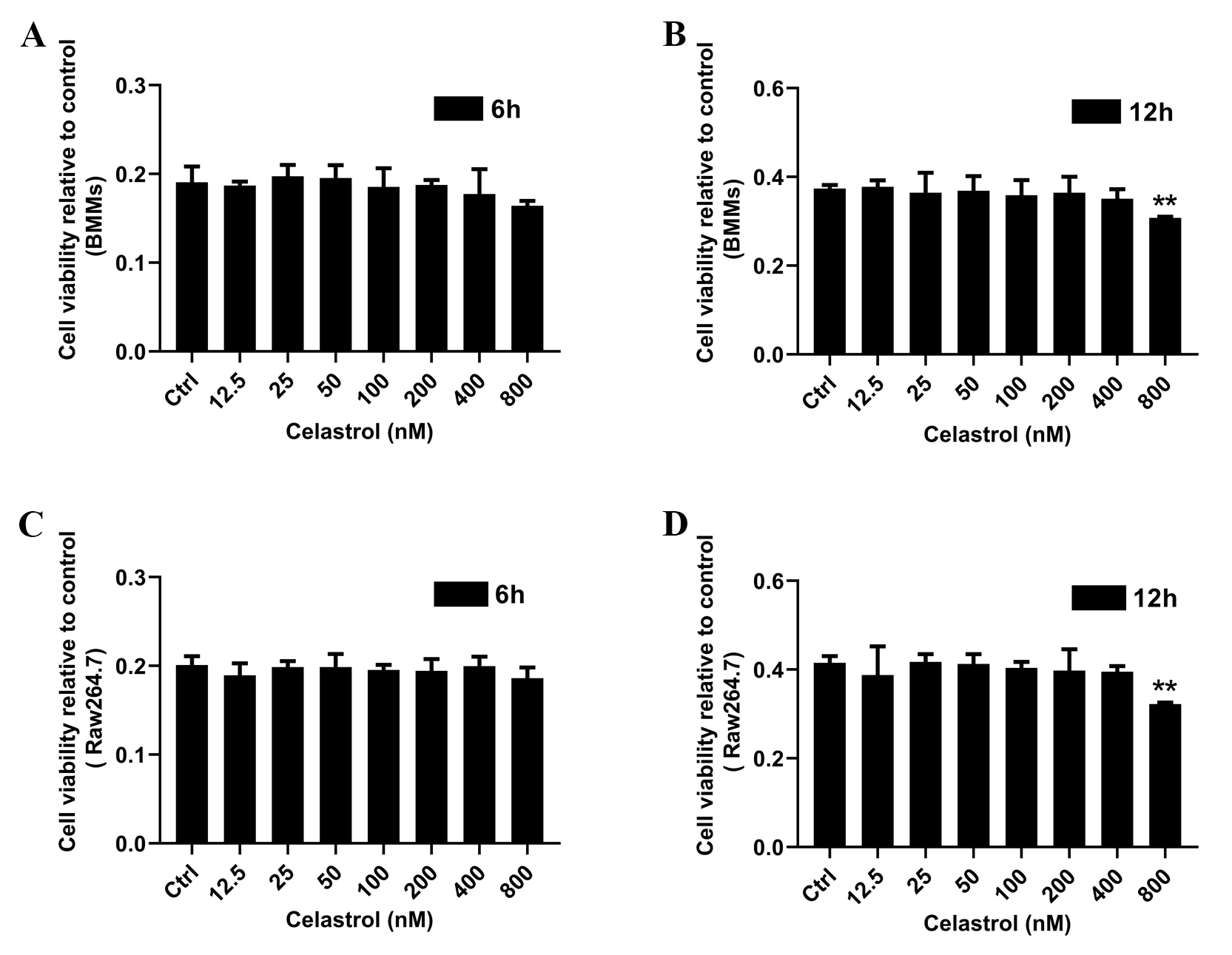

Supplement: Supplementary file 1 [file Image1.tif]
